# Interoceptive Ability Predicts Survival on a London Trading Floor

**DOI:** 10.1038/srep32986

**Published:** 2016-09-19

**Authors:** Narayanan Kandasamy, Sarah N. Garfinkel, Lionel Page, Ben Hardy, Hugo D. Critchley, Mark Gurnell, John M. Coates

**Affiliations:** 1 Institute of Metabolic Science and National Institute for Health Research Cambridge Biomedical Research Centre, University of Cambridge, Addenbrooke’s Hospital, Cambridge CB2 0QQ, UK; 2 Brighton and Sussex Medical School and Sackler Centre for Consciousness Science, University of Sussex, Brighton BN1 9RR UK; 3 Queensland University of Technology, Business School, Brisbane, QLD 4001, Australia; 4 University of Cambridge, Judge Business School, Cambridge CB2 1AG, United Kingdom

## Abstract

Interoception is the sensing of physiological signals originating inside the body, such as hunger, pain and heart rate. People with greater sensitivity to interoceptive signals, as measured by, for example, tests of heart beat detection, perform better in laboratory studies of risky decision-making. However, there has been little field work to determine if interoceptive sensitivity contributes to success in real-world, high-stakes risk taking. Here, we report on a study in which we quantified heartbeat detection skills in a group of financial traders working on a London trading floor. We found that traders are better able to perceive their own heartbeats than matched controls from the non-trading population. Moreover, the interoceptive ability of traders predicted their relative profitability, and strikingly, how long they survived in the financial markets. Our results suggest that signals from the body - the gut feelings of financial lore - contribute to success in the markets.

Physiologists often use the term ‘gut feeling’ as a colloquial synonym for any interoceptive sensation that guides behaviour. These sensations carry viscerosensory information and may originate from many tissues of the body, the heart and lungs, for instance, not just the gut. Interoception differs from exteroception, which is the sensing of the outside world through sight, sound, touch, etc.; and from proprioception, which is the sensing of the body’s position. Interoceptive signals report on the visceromotor, inflammatory, and thermoregulatory state of the body; and they engender somatic feelings such as breathlessness, racing heart, thermal stress, as well as fullness from gut, bladder, and bowel^1^,^2^,^3^,^4^,^5^. By reporting on the homeostatic state of the body^6^,^7^,^8^,^9^ these signals underpin motivational states such as hunger, thirst, pain, and anxiety^10^,^11^,^12^,^13^.

Interoceptive information, even if unconscious or occupying the fringes of awareness, provides valuable inputs in risky decision making. Antoine Bechara and Antonio Damasio have observed that high-risk choices made on the Iowa Gambling Task are accompanied by rapid and subtle physiological changes – for example, sympathetic skin response - which they call ‘somatic markers’^14^. Such autonomic responses feedback on the brain to bias our decisions, steering us away from gambles with negative expected returns and towards ones with positive expected returns. People can generate somatic markers and make optimal decisions on gambling tasks before they are able to articulate the reasons for their decisions^15^. Moreover, without somatic markers valancing the available options people can become lost among the possible choices and be unable to choose in an optimal manner. For example, patients with damage to the ventromedial prefrontal cortex can lose the coupling of bodily arousal with decisions; and even though they maintain normal intellect they suffer a recurrent failure to learn from negative feedback^16^. Gut feelings are thus valuable guides when taking risks.

People vary in their ability to generate and sense interoceptive signals, an ability that can be measured by various physiological tests, the most common being heartbeat detection^17^,^18^,^19^,^20^,^21^. Studies have found that higher scores on heartbeat detection predict superior performance on some laboratory gambling tasks^22^,^23^,^24^,^25^. However, little field research has been conducted to determine if the benefits of interoceptive sensitivity predicted by the somatic marker hypothesis have real-world implications. Do individual differences in interoceptive ability predict performance in actual high-stakes risk taking? Traders and investors in the financial markets frequently talk of the importance of gut feelings for selecting profitable trades. We therefore conducted a study on the trading floor of a mid-sized hedge fund in the City of London to test the hypothesis that traders with a greater ability to sense signals from their bodies do in fact make more money.

## Methods

We recruited 18 male traders engaged in high frequency trading. What that means is that they bought and sold futures contracts but held their trading positions for only a short period of time, seconds or minutes, a few hours at the most. This form of trading requires an ability to assimilate large amounts of information flowing through news feeds, to rapidly recognize price patterns, and to make large and risky decisions with split-second timing.

These traders did not deal with clients, so did not benefit from the margins earned on customer business. Furthermore, each of the traders had equal access to information and capital, so their performance (i.e. profitability) was determined purely by skill. They received no year-end bonus based on the performance of the firm as a whole; their individual compensation consisted entirely of a share of their trading profits. This niche of the financial markets is particularly unforgiving, and selection acts quickly: while successful traders may earn in excess of £10 million per year, unprofitable ones do not survive for long. We monitored these traders during a particularly volatile period (the tail end of the European sovereign debt crisis) so the performance of each trader reflected his ability to make money during periods of extreme uncertainty.

We measured individual differences in each trader’s capacity to detect interoceptive signals - i.e. the changes, often subtle, in the physiological state of their bodies – by means of two established heartbeat detection tasks. These tasks quantify how accurately a person, when at rest, can feel their heart beating.

Each trader first performed a heartbeat counting task, in which he was instructed to silently count, without touching chest or any pulse point, how many heartbeats he felt over brief time intervals^19^. This counting task was repeated six times, using randomized time periods of 25, 30, 35, 40, 45 and 50 seconds. The number of heartbeats perceived by the trader within each time period was compared with the actual number of heartbeats, as recorded over that interval objectively and non-invasively by pulse oximetry. A heartbeat detection accuracy score was expressed as a percentage using the following equation^26^:

Heartbeat detection score = ([1 − [absolute value of (actual − estimated) ÷ mean of (actual + estimated)]) × 100.

After each trial of the counting task, the traders rated their confidence in their heartbeat detection using a visual analogue scale. At one end of this scale was marked “Total guess/No heartbeat awareness”, at the other end “Complete confidence/Full perception of heartbeat”. Four traders had a single outlying counting score (identified by the Tukey method of interquartile ranges) on one of the six counting trials. For each of these four traders, this reading was dropped and their final score was averaged from the remaining five trials.

In the second heartbeat detection task traders were asked to judge if a series of ten auditory tones was played synchronously with, or delayed relative to, their own heartbeats^20^,^27^. The tones (presented at 440 Hz and lasting 100 ms) were triggered by individual heartbeats. On synchronous trials, the ten notes occurred at the rising edge of finger pulse pressure wave; on delay trials they followed 300 ms later. At the end of each trial, the participant indicated whether the tones were synchronised or not. Each trader performed 15 trials of this task. Their score was the percentage of right answers. This task was performed after the counting task to prevent the traders from developing any explicit knowledge about their own heartrates^28^.

Data were also collected on each trader’s age, years of trading, and profit and loss statement, known as their P&L. The study was approved by the ethics committee of the School of Biological Sciences at the University of Cambridge. Comparative data from a control group of 48 high-functioning (mostly students and postgraduates) non-trader males, matched with traders on age, were drawn from participants who had performed identical interoceptive tests (same equipment and procedures) in studies undertaken at the University of Sussex^29^ and approved by the Brighton and Sussex Medical School Research Governance and Ethics Committee. The experiment was conducted according to the code of ethics on human experimentation established by the Helsinki declaration and in accordance with the approved guidelines. All participants provided written informed consent.

For data analysis we employed robust single linear regression: p values were computed from the t statistics associated with the coefficients using Huber-White robust standard errors, adjusted for small sample sizes, which give estimates robust to heteroskedasticity.

## Results

### Traders have enhanced interoceptive ability compared to non-trading individuals

**Figure 1:**
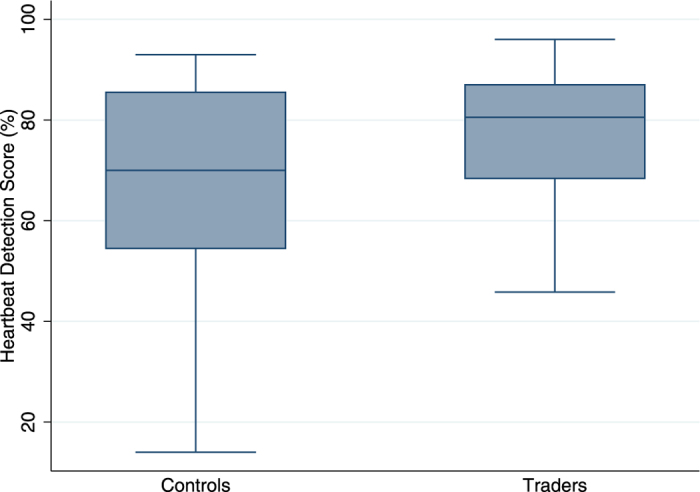
Box plots showing that mean interoceptive accuracy (score on heartbeat counting task) for traders (N = 18) was significantly higher than for a cohort of non-traders (N = 48).

As a first step in assessing the importance of interoception in financial risk taking, we tested simply if traders scored higher on heartbeat detection tasks than non-trading controls. We compared the average score on the heartbeat counting task for the traders with the average score from our control group. We found that the traders had significantly higher scores, indicating greater interoceptive accuracy, than the controls, with a mean score of 78.2 for traders and 66.9 for controls (p = 0.011, N = 66, Fig. 1).

### Interoceptive ability predicts trader profitability

**Figure 2:**
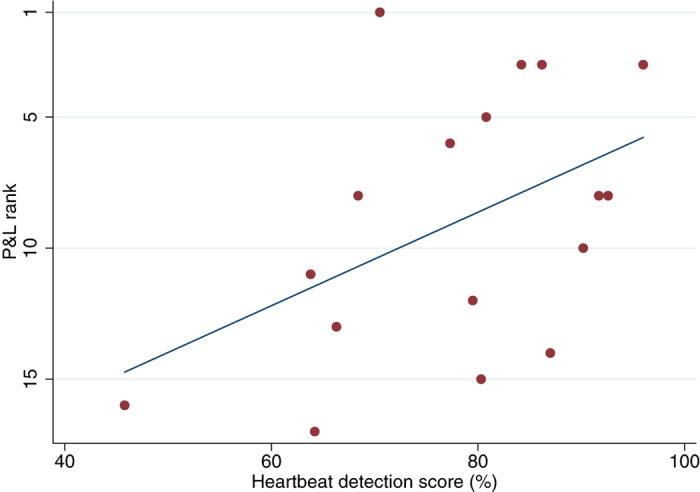
Regression line plotting score on the heartbeat counting task against the traders’ rank ordered P&L, with 1 representing the most profitable trader, 17 the least.

To test the hypothesis that interoceptive accuracy predicts trading performance, we collected data on each trader’s average daily profit and loss (P&L) over the previous year. One of the traders was acting as trading manager, so did not have his own P&L; another trader had a P&L more than 5 standard deviations higher than the mean of other traders, so even with data transformations he distorted the distribution. Both traders were therefore omitted from analyses that used ‘raw’ P&L. We found that the traders’ scores on the heartbeat counting task predicted their P&L (coeff = 2.61, R^2^ = 0.27, p = 0.007, N = 16). To further validate this association, we also rank-ordered the P&L, which permitted the inclusion of the trader with the outlying P&L. Again we found that heartbeat detection score predicted relative trading performance (coeff = 17.84, R^2^ = 0.22, p = 0.01, N = 17, Fig. 2).

### Interoceptive ability predicts survival in the financial markets

**Figure 3:**
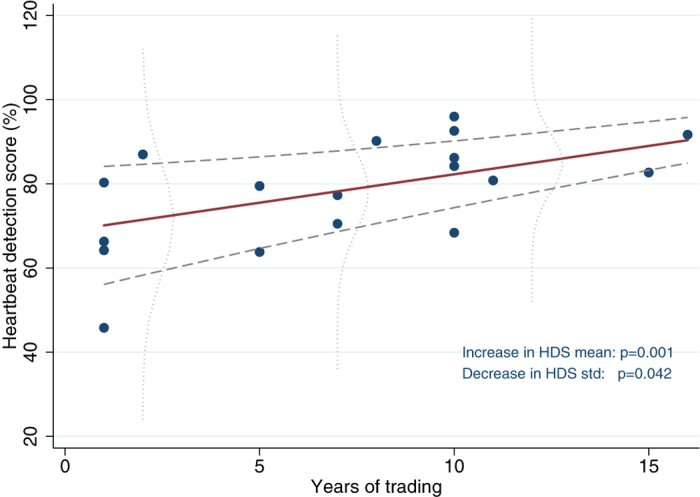
Years of trading experience plotted against heartbeat detection score (HDS). Solid red line is regression plot. A regression model with conditional mean and conditional standard deviation (std) estimated jointly is used to assess the significance of changes in the heartbeat detection mean and std over years of trading. Light dashed horizontal lines are +/−1 std. Vertical dotted lines show distributions of residuals for each bucket of trading experience. These distributions show a declining variance of heartbeat detection as years of experience increase.

If heartbeat detection score predicts traders’ P&L, does it also predict how long traders survive in the financial markets? To answer this question we plotted heartbeat detection scores against years of experience in the financial markets and found that a trader’s heartbeat counting score predicted the number of years he had survived as a trader (coeff = 21.64, R^2^ = 0.344, p = 0.001, N = 18, Fig. 3).

Does this result mean the markets select for traders with greater interoceptive ability? To pursue this analysis we reasoned as follows: i) If firms know nothing about interoception (and the hosting firm of our study did not) they will not hire traders on the basis of heartbeat detection scores. We should find, therefore, that beginner traders and the non-trading population have mean heartbeat detection scores and a standard deviation of scores that do not differ significantly. ii) If the market selects for traders with good gut feelings (i.e. high heartbeat detection scores), then what we should find is that as the traders’ careers progress and the market selects for traders with better gut feelings, those with low heartbeat detection scores will be eliminated from the markets. Average heartbeat detection score for the traders will rise, while the standard deviation of scores will fall as the lower end of the range drops out.

To test this possibility, we partitioned the group according to experience: junior traders (1–4 yrs experience), mid (5–8 yrs), and senior (>8 yrs) and then calculated the mean and standard deviation of detection scores for each bucket (Table 1). We found that beginner traders did not differ significantly from controls in the mean (p = 0.852, N = 53) nor standard deviation (p = 0.614, N = 53) of their heartbeat detection score. Over time, however, the traders’ mean heartbeat detection score did indeed rise, from 68.7 for beginners to 85.3 for experienced traders. This latter level differed significantly from controls (T test, p = 0.02, N = 56). In addition, the standard deviation decreased, from 16 for beginners to 8.6 for experienced traders, and this latter standard deviation also differed significantly from controls (F test, p = 0.018, N = 56) (Table 1). This result is represented graphically in Fig. 3, where the variance of residuals declines with the number of years of trading (coeff = −0.063, p = 0.042, N = 18), the pattern of these residuals forming a wedge shape rather than the customary parallelogram.

### Synchronization task demonstrates a bi-modal distribution

We found, as have others, that the data from the heart beat synchronization task (asking if a series of tones was synchronised to heartbeat) produced a bimodal distribution: a few subjects excelled, with scores around the 90 level, while the remainder had scores tightly clustered around the 60 level^30^,^31^. It is thought that the synchronisation task produces this bimodal distribution because it involves a more complex and difficult integration of interoceptive (heartbeat) signals with exteroceptive (audible tone) signals and crossmodal timing. This task may help identify high scoring individuals from relatively large populations. But in our sample we found no correlations between the traders’ scores on the synchronization task and their scores on the counting task; nor between the synchronization task and any of our dependent variables.

### Interoceptive accuracy but not confidence informs trading performance

We next analysed the traders’ level of confidence in their estimates of their own heartbeats, as recorded on visual analogue scales during the heartbeat counting task^29^,^32^. We found no significant correlations between confidence and heartbeat detection accuracy (coeff = 0.17, p = 0.51, N = 17); nor between confidence and P&L (coeff = −0.01, p = 0.97, N = 17); nor between confidence and years of survival (coeff = −0.16, p = 0.54, N = 17).

This disjunct between the traders’ heartbeat detection accuracy and their confidence in their accuracy may seem contradictory but, as with many physiological measures, objective performance and subjective appraisal often diverge. In the case of interoceptive ability, it is conceivable that a self-conscious awareness of interoceptive signals impairs the signal’s utility because people may dismiss the signals as ‘merely’ physical and distracting. Alternatively, self-consciousness may impair risk taking in much the same way that focusing self-consciously on, say, your tennis stroke can impair your game. Related to this point, it is noteworthy that the control group in our study included 16 medical students who, despite their more advanced understanding of the cardiovascular system, performed on average worse than the traders on the counting task (65.9 versus 78.2), although this difference only approached significance (p = 0.056, N = 34).

### Physiological markers predict heartbeat detection score

If heartbeat detection ability predicts profitability and survival in the markets, can we offer any insights into what predicts heartbeat detection ability itself? To address this question we examined physiological data that was available for a subset of traders (N = 14). In a multiple regression model predicting heartbeat detection score, we found that a lower body mass index (BMI), a lower heart rate (HR), and a lower root mean square of successive differences (RMSSD) in R-R heartbeat intervals (a measure of heart rate variability) predicted higher heartbeat detection scores: R^2^ = 63.7%, p = 0.006; HR (coef = −0.01, p = 0.012); BMI (coef = −0.11, p = 0.003); RMSSD (coef = −0.006, p = 0.009)^33^,^34^,^35^,^36^. Previous research has found that heartbeat detection scores decline with age^37^, but we found that age was not a significant predictor.

Given how strongly the physiology predicted heart beat detection scores (HBD), the question then arose: does the physiology itself predict the P&L and years of survival, independently of heart beat detection? To answer this question we combined all available independent variables (HBD, HR, RMSSD, BMI) in a multiple regression model and then employed Akaike Information Criteria to select the variables that best explain P&L and years of survival. The Akaike Information Criterion measures the quality of a model as a trade-off between its goodness of fit (including R^2^ and p value) and its complexity, i.e., number of variables.

We found that heart beat detection and physiology jointly predicted P&L and years of survival. Specifically, P&L was best predicted by a model which included heart beat detection and a single physiological variable, heart rate variability: R^2^ = 45.4%, p = 0.008; HBD (coeff = 16.08, p = 0.011); RMSSD (coeff = −0.09, p = 0.019). Years of survival was predicted by the same two variables: R^2^ = 60.5%, p = 0.001; HBD (coeff = 15.5, p = 0.004); RMSSD (coeff = −0.12, p = 0.001). The other variables that predicted heart beat detection scores (HR, BMI) did not help predict either P&L nor years of survival, and may therefore have their main effects on these dependent variables through their intermediate effects on interoception. The results of this analysis suggest that the physiological variables we examined affect P&L and survival partly through their effects on interoception and partly through their direct effects.

## Discussion

Traders in the financial world often speak of the importance of gut feelings for choosing profitable trades. By this they mean that subtle physiological changes in their bodies provide cues helping them rapidly select from a range of possible trades the one that just ‘feels right’. Our findings suggest that the gut feelings informing this decision are more than the mythical entities of financial lore - they are real physiological signals, valuable ones at that.

In our study we sought to determine if the benefits of interoceptive sensitivity suggested by previous research could be verified in a field study of high stakes risk taking. Using a heartbeat detection test with real traders we found evidence suggesting that interoceptive sensitivity does indeed contribute to profitable risk taking in the financial markets. We found that heartbeat detection scores among the traders were significantly higher than those found among a matched sample of non-trading controls (Fig. 1), and that heartbeat detection scores predicted the traders’ relative P&L (Fig. 2) and the length of time they had survived in the markets (Fig. 3). We further found that the heartbeat detection scores were themselves predicted by the traders’ heart rates, heart rate variability, and BMI.

Our study, being field work, could not establish causation. Our results may be consistent with recent laboratory research showing that heartbeat detection skills predict more effective risk taking^22^,^23^,^24^,^25^, but there could be other interpretations. For example, some researchers have found that heartbeat detection ability increases during stress^38^, so it could be argued that heartbeat detection skills correlated with years of survival merely because experienced traders, taking larger risks, are subjected to greater stresses. However, in trading, as in many other professions, experienced and successful individuals, being more in control, are commonly less stressed than beginners. Certainly, we found no significant increase in levels of heart rate or the stress hormone cortisol in experienced traders when compared with their less experienced counterparts (data not shown). We did find that heart rate variability was lower in experienced traders, as reported above, but we interpret this as a perfectly healthy and effective state of arousal in active traders - much as one observes in elite athletes - rather than a sign of distress.

Alternatively, it could be argued that success in trading is due to some unobserved variable, perhaps mere luck, and as the careers of profitable traders mature they can afford to allocate more time to activites contributing to physical fitness, such as sports, gym, and yoga. Their fitness improves, BMI and baseline heart rate drop, and as a result their heartbeat detection ability improves. Here trading success leads to an improvement in physiological fitness which in turn leads to an improvement in heartbeat detection skill. This interpretation is credible. However, arguing against it, and equally against the above interpretation relying on stress levels, is the fact that the physiology does not independently predict P&L and years of survival. Even when controlling for BMI, heart rate and heart rate variability, we find that heartbeat detection skill remains as a significant independent predictor of P&L and years of survival.

Furthermore, we formulated our hypothesis and interpreted our study results so as to build on existing and cumulative evidence that suggests interoceptive sensitivity contributes to effective risk taking, and on the known neural mechanisms underlying this evidence^39^,^40^. Interoceptive signals are communicated along a dedicated lamina-1 spinothalamocortical pathway as well as the vagus nerve to ‘interoceptive centres’ in the somatosensory, anterior insular, and ventromedial prefrontal cortices^2^,^3^,^6^. A recent study involving a gambling task has found that one of these interoceptive integration sites - the insula – showed increased activation when study participants exited a profitable trade^39^.

Our findings present anomalous data for the influential Efficient Markets Hypothesis of economic theory. According to strong versions of this hypothesis, the market is random, meaning that no trait or skill of an investor or trader – not their IQ, education, nor training - can improve their performance, any more than these traits and skills could improve their performance at flipping coins. We find on the contrary that the physiological state of traders does have large effects on their success and survival^41^,^42^,^43^. Such a finding has profound implications for the understanding of financial markets, specifically by reorienting attention away from risk takers’ psychological traits towards their physiological ones^44^.

More generally, our results suggest that economics and the behavioural assumptions upon which it rests, will benefit from a greater involvement with human biology. Today there is a debate between, on the one hand, classical economists who argue that economics has no use for the findings of psychology and neuroscience^45^, and on the other, behavioural economists who do draw lessons from these experimental sciences^46^,^47^. What has been missing from this debate is evidence concerning the role of somatic signals, in other words, the body, in guiding our decision-making and behaviour and, crucially, our risk taking.

## Additional Information

**How to cite this article**: Kandasamy, N. *et al*. Interoceptive Ability Predicts Survival on a London Trading Floor. *Sci. Rep.* **6**, 32986; doi: 10.1038/srep32986 (2016).

## Figures and Tables

**Table 1:** Mean and standard deviation of detection scores for controls and for traders organized by years of experience.

Cohort	Detection Mean	Difference from controls	Detection Std	Difference from controls
Controls (N = 48)	66.9		21.3	
Junior Traders 1–4 yrs (N = 5)	68.7	+1.8 (p = 0.852, N = 53)	16.0	−5.3 (p = 0.614, N = 53)
Mid Traders 5–8 yrs (N = 5)	76.3	+9.4 (p = 0.339, N = 53)	9.9	−11.4 (p = 0.144, N = 53)
Senior Traders >8 yrs (N = 8)	85.3	+18.4 (p = 0.02, N = 56)	8.6	−12.7 (p = 0.018, N = 56)

T tests are used to compare means, and F tests of equality of standard deviation are used to compare standard deviations.
